# Impact of TyG index on coronary plaques in patients with coronary artery disease under aggressive lipid-lowering therapy

**DOI:** 10.3389/fendo.2026.1766778

**Published:** 2026-02-12

**Authors:** Tatsuya Fukase, Tomotaka Dohi, Norihito Takahashi, Shinichiro Doi, Iwao Okai, Hiroshi Iwata, Shinya Okazaki, Katsumi Miyauchi, Hiroyuki Daida, Tohru Minamino

**Affiliations:** 1 Department of Cardiovascular Biology and Medicine, Juntendo University Graduate School of Medicine, Tokyo, Japan; 2 Department of Radiological Technology, Faculty of Health Science, Juntendo University Graduate School, Tokyo, Japan; 3 Japan Agency for Medical Research and Development-Core Research for Evolutionary Medical Science and Technology (AMED-CREST), Japan Agency for Medical Research and Development, Tokyo, Japan

**Keywords:** coronary atheroma volume, lipid-lowering therapy (LLT), percutaneous coronary intervention (or PCI), serial intravascular ultrasound, triglyceride-glucose index (TyG index)

## Abstract

**Background:**

One of residual risks of coronary artery disease (CAD) occurrence and coronary plaque progression might be a triglyceride-glucose index (TyG index). Thus, this study aimed to investigate the impact of TyG index on coronary artery plaques detected by serial intravascular ultrasound (IVUS) in patients with CAD under low-density lipoprotein cholesterol (LDL-C) lowering therapy represented by statins.

**Methods:**

This observational cohort study included three clinical prospective trials (the ENTERPRISE trial, ESPECIAL-ACS study, and ZEUS trial) in which coronary plaques were assessed using serial grayscale IVUS at baseline and at 6–12 months follow-up. The patients were divided into two groups according to the cutoff value of baseline TyG index (dichotomized to ≥ or <8.5) based on a previous report. The primary endpoint was defined as an absolute change in percentage atheroma volume (PAV) from baseline to follow-up.

**Results:**

A total of 131 patients (mean age: 63 years; 92% men) completed the study and were analyzed. Compared to the low TyG index group, the high TyG index group had higher body mass index, triglyceride, and proportion of male, diabetes mellitus, and smoking history, as well as lower high-density lipoprotein cholesterol; whereas, there was no difference in LDL-C between both groups. The absolute change in PAV between the low TyG index group and high TyG index group only in patients who received aggressive lipid-lowering therapy (LLT) was −3.5±1.4% by analysis of covariance (*p*=0.017). In addition, the baseline TyG index, male, and %change in LDL-C were significantly associated with the absolute change in PAV under aggressive LLT by multiple linear regression analysis.

**Conclusions:**

An elevated TyG index could be a residual risk of coronary plaque progression under aggressive LLT.

## 1 Introduction

The current guidelines on lipid-lowering therapy (LLT) recommend an aggressive lipid management to reduce cardiovascular disease risk (1, 2). In particular, low-density lipoprotein cholesterol (LDL-C)-lowering therapy plays the most important role in the prevention of cardiovascular events, and high-intensity statin therapy has contributed to the reduction of cardiovascular event rate (3), as well as the regression of atherosclerotic coronary plaques (4, 5). In addition, coronary plaque regression caused by LLT is associated with a reduced cardiovascular risk (6); however, residual risks of cardiovascular events and coronary plaques persist despite aggressive LLT. One of these risks might be a triglyceride-glucose index (TyG index), and the TyG index was first proposed in 2008 as an alternative biomarker for insulin resistance, due to simple calculation using fasting triglyceride and fasting plasma glucose levels, and few constraints on time and cost (7). The TyG index is closely related to different systemic diseases, indicating the role of metabolic parameters in various diseases; in particular, there are many reports on the significant association between the TyG index and coronary artery disease (CAD) (8–11). Additionally, the relationship between the TyG index and coronary plaque characteristics has been recently reported using optical coherence tomography and coronary computed tomography angiography (12–14). The TyG index has been attracting attention not only at the patient level but also at the plaque level; thus, the aim of this study was to investigate the impact of the TyG index on coronary artery plaques detected by serial intravascular ultrasound (IVUS) in patients with CAD under LLT represented by statins.

## 2 Materials and methods

### 2.1 Ethics statements

The prospective registry database of patients who underwent any percutaneous coronary intervention at Juntendo University Hospital, Tokyo, Japan (Juntendo Physicians’ Alliance for Clinical Trial: J-PACT) is publicly registered (University Medical Information Network Japan-Clinical Trials Registry, UMIN-CTR 000035587). In addition, the ethics committee of the Juntendo Clinical Research approved this study (reference number E22-0409), and all participants provided written informed consent or opting-out consent. The study was conducted in accordance with the principles of the Declaration of Helsinki (15).

### 2.2 Study design and population

This was an observational cohort study that included statin-treated patients who participated in three clinical prospective trials that evaluated the impact on coronary plaque burden measured by serial IVUS at baseline and 6 to 12 months later. Overall, this study enrolled 141 patients with stable CAD and acute coronary syndrome (ACS) from the following trials: (1) the ENTERPRISE trial assessed continuous positive airway pressure in patients with stable CAD coexisting with sleep-disordered breathing (16), (2) the ESPECIAL-ACS study assessed dipeptidyl peptitase-4 inhibitors in patients with ACS and diabetes mellitus (DM) (17), and (3) the ZEUS trial assessed the combination of statin and ezetimibe in patients with ACS (18). The following patients were excluded from this study: patients with severe obesity [body mass index (BMI) ≥35.0 kg/m^2^], patients for whom LDL-C-lowering therapy including statins was not performed, patients without data of the baseline TyG index, and patients for whom sufficient analysis data were not available.

### 2.3 Data collection and definitions

Regarding lipid profiles, high-density lipoprotein cholesterol (HDL-C), LDL-C, and triglyceride levels were assayed using LABOSPECT 008α (Hitachi, Ltd., Tokyo, Japan). The Japan Atherosclerosis Society has recently updated the lipid management for secondary prevention in atherosclerotic cardiovascular disease (19). Thus, lipid control in this study was defined as aggressive LLT if the following target LDL-C values were achieved: on-statin treatment LDL-C value <55 mg/dL in patients with ACS or DM, on-statin treatment LDL-C value <70 mg/dL in patients with stable CAD and non-DM, or on-statin treatment LDL-C value decreased by ≥50% from baseline; on the other hand, lipid management was defined as non-aggressive LLT when these target values of LDL-C were not achieved. Patients with a blood pressure >140/90 mmHg or those receiving antihypertensive drugs were considered hypertensive (20). Patients with a hemoglobin A1c (HbA1c) level of ≥6.5%, those administered with oral hypoglycemic agents, or those receiving insulin injection therapy were defined as having DM (21). A family history of premature CAD was defined as the presence of any first-degree relative with premature CAD (age <55 years for men and <65 years for women) (22). Chronic kidney disease was defined as an estimated glomerular filtration rate of <60 mL/min/1.73 m^2^ based on the Modification of the Diet in Renal Disease equation modified using the baseline serum creatinine level (23).

The formula for calculating the TyG index is TyG index = Ln [fasting triglyceride (mg/dL) × fasting plasma glucose (mg/dL)/2]. The relationship between the TyG index and cardiovascular events in Southeast Asia has been reported in recent years, and the cutoff value of the TyG index predicting cardiovascular events is 8.3 to 8.5 (24–27). In this study, the patients were divided into two groups according to the cutoff value of the baseline TyG index (dichotomized to ≥ or <8.5), based on a large-scale clinical trial using a database of the National Health and Nutrition Examination Survey (28).

### 2.4 IVUS imaging acquisition and analysis

A non-culprit lesion segment was defined as the proximal or distal site to the culprit lesion segment except for the 5-mm proximal and distal stent edges, and were imaged using grayscale IVUS and near-infrared spectroscopy and IVUS (NIRS-IVUS) pullback at both baseline and 6–12 months later. Grayscale IVUS and NIRS-IVUS were performed using the commercially available systems Altantis SR Pro2 (Boston Scientific, Natick, MA, USA), ViewIT (Terumo, Tokyo, Japan), and TVC Imaging System or Makoto Imaging System (Infraredx, Bedford, MA, USA). A grayscale IVUS and NIRS-IVUS catheter were inserted distal to the non-culprit lesion and pulled back at a rate of 0.5 mm/s, after intracoronary injection of nitroglycerin.

Quantitative grayscale IVUS measurements were performed using the Netra 3D iVUS system (AcImage, CA USA), VISIATRAS (Terumo, Tokyo, Japan), and QIvus version 2.1 (Medis, Leiden, the Netherlands) to quantify plaque volume according to two clinical expert consensus documents (29, 30). Baseline and follow-up IVUS images were reviewed side-by-side on a display, and target segments were selected. The quantitative IVUS analysis was accurately performed off-line by the cardiologists who were blinded to the clinical data. The lumen cross-sectional area (CSA) (31) and external elastic membrane (EEM) CSA were measured every frame; in addition, this software can measure vessel volume, and percentage atheroma volume (PAV) (%) = [∑ (EEM CSA − lumen CSA)/∑ EEM CSA] × 100.

### 2.5 Statistical analysis

All data were analyzed using JMP^®^ Pro, version 16.0.0 for Macintosh (SAS Institute, Cary, NC, USA). All probabilities were expressed as two-tailed values, with statistical significance set at *p* < 0.05. All confidence intervals (CIs) were computed at 95% level.

Categorical data were presented as number (percentage) and were compared using the chi-square test. Continuous variables were expressed as mean ± standard deviation or median (interquartile range) and compared using a one-way analysis of variance or the Kruskal–Wallis test. The Shapiro–Wilk test was used to examine whether the scores were likely to follow a certain distribution in all patients. If *p* < 0.05, the variables were not considered normally distributed. Univariable analysis of the relationship between the absolute change in PAV and the baseline TyG index was performed using the Pearson correlation analysis. The comparison of absolute change in PAV between the low-TyG-index group and high-TyG-index group was evaluated using analysis of covariance. Multivariable linear regression analyses of the absolute change in PAV and %change in the TyG index were performed to evaluate the predictors. A sensitivity analysis was performed to analyze how the value of the TyG index has affected the endpoints, and this study investigated whether similar trends were obtained within the TyG index range of 8.3 to 8.5 shown in the aforementioned reports (24–27).

## 3 Results

### 3.1 Patients’ clinical characteristics

**Figure 1:**
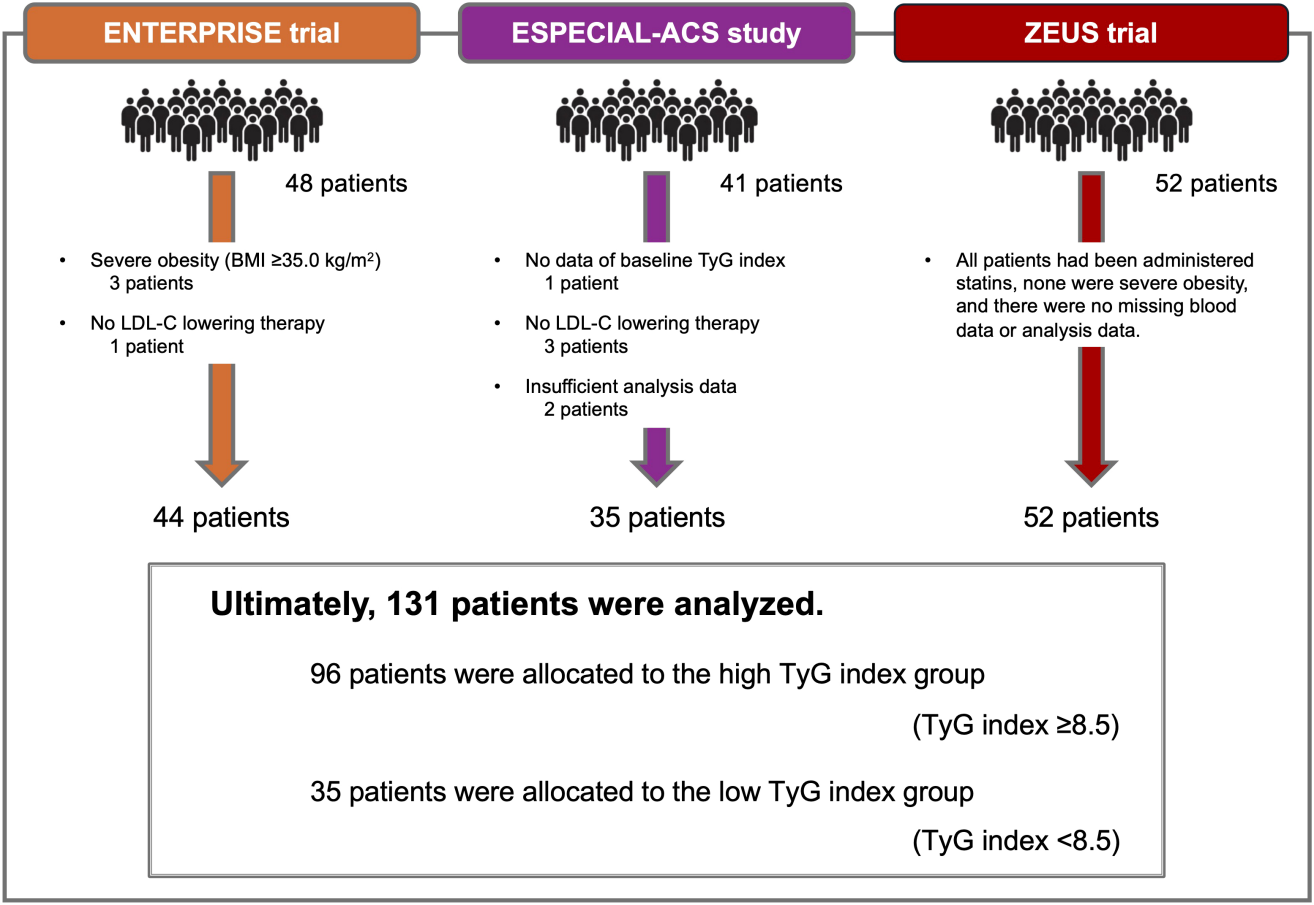
Study flowchart. The ENTERPRISE trial enrolled 48 patients with stable coronary artery disease coexisting with sleep-disordered breathing, and excluded three patients with severe obesity (BMI ≥35 kg/m^2^) and one patient who was not receiving LDL-C-lowering therapy at baseline. The ESPECIAL-ACS study enrolled 41 patients with acute coronary syndrome and diabetes mellitus, and excluded 1 patient who did not have baseline TyG index, 1 patient who was not receiving LDL-C-lowering therapy at baseline, and 2 patients who had insufficient analysis data. The ZEUS trial enrolled 52 patients with acute coronary syndrome, and no patients met the exclusion criteria. Ultimately, 131 patients were analyzed, and these patients were divided into two groups according to the cutoff value of baseline TyG index (dichotomized to ≥ or <8.5) based on a previous report. A total of 96 patients were allocated to the high-TyG-index group, and 35 patients were allocated to the low-TyG-index group. BMI, body mass index; LDL-C, low-density lipoprotein cholesterol; TyG index, Triglyceride-glucose index.

Among 141 patients, 3 had severe obesity, 4 were not receiving LLT at baseline, 1 patient did not have a baseline TyG index, and 2 had insufficient analysis data. Ultimately, 131 patients completed the study, and their data were analyzed. According to the cutoff value of the baseline TyG index, the patients were divided into two groups; 96 patients were allocated to the high-TyG-index group (TyG index ≥8.5) and 35 patients were allocated to the low-TyG-index group (TyG index <8.5), as shown in **Figure 1**.

The baseline clinical characteristics of the patients are summarized in **Table 1**. The mean patient age was 63 ± 10 years, and 92% of the patients were men. Overall, hypertension was observed in 78%, DM in 59%, smoking history in 77%, and family history of premature CAD in 22%. Compared to the low-TyG-index group, the high-TyG-index group had higher BMI, triglyceride, fasting plasma glucose, HbA1c, and proportion of men, DM, and smoking history, as well as lower HDL-C, whereas there was no difference in LDL-C between both groups. Overall, the mean TyG index was 8.8 ± 0.6 at baseline and significantly rose to 9.4 ± 0.6 during the follow-up period regardless of the high-TyG-index group and the low-TyG-index group (*p* < 0.001).

**Table 1:** Baseline patients’ characteristics.

Variables	Overall (*n* = 131)	High-TyG-index group (*n* = 96)	Low-TyG-index group (*n* = 35)	*P*-value
Age, years	63 ± 10	63 ± 10	65 ± 9	0.227
Male, *n* (%)	120 (92)	91 (95)	29 (83)	0.041
Body mass index, kg/m^2^	25.0 ± 3.5	25.4 ± 3.4	24.0 ± 3.8	0.040
Hypertension, *n* (%)	102 (78)	76 (80)	26 (74)	0.488
Diabetes mellitus, *n* (%)	77 (59)	62 (65)	15 (43)	0.026
Smoking, *n* (%)	95 (77)	75 (84)	20 (59)	0.004
Family history of premature CAD, *n* (%)	29 (22)	23 (24)	6 (17)	0.381
TyG index	8.8 ± 0.4	9.1 ± 0.5	8.1 ± 0.3	<0.001
Triglyceride, mg/dL	132 ± 66	155 ± 76	68 ± 18	<0.001
HDL-C, md/dL	45 ± 11	43 ± 10	51 ± 14	0.001
LDL-C, md/dL	101 ± 30	104 ± 32	94 ± 24	0.099
Fasting plasma glucose, mg/dL	124 ± 34	132 ± 38	103 ± 20	<0.001
HbA1c, %	6.5 ± 0.8	6.6 ± 0.8	6.3 ± 0.7	0.015
Hs-CRP, mg/dL	0.30 (0.05, 0.99)	0.27 (0.05, 1.03)	0.45 (0.09, 0.98)	0.458
Achieved aggressive LLT, *n* (%)	59 (46)	39 (41)	20 (59)	0.074
Acute coronary syndrome, *n* (%)	87 (66)	65 (68)	22 (63)	0.605

CAD, coronary artery disease; HbA1c, hemoglobin A1c; HDL-C, high-density lipoprotein cholesterol; Hs-CRP, high-sensitivity C-reactive protein; LDL-C, low-density lipoprotein cholesterol; LLT, lipid-lowering therapy; TyG index, triglyceride-glucose index.

### 3.2 Coronary plaque characteristics

IVUS measurements showed that there was an overall tendency for coronary plaque regression as shown in **Table 2**; the mean absolute change in PAV was −1.2% in the median non-culprit lesion length of 12 mm. There was no significant difference in the mean absolute change in PAV between the high-TyG-index group and the low-TyG-index group, although the high-TyG-index group had a significantly higher mean PAV at the time of follow-up, compared to the low-TyG-index group.

**Table 2:** Intravascular ultrasound measurements.

Variables	Overall (*n* = 131)	High-TyG-index group (*n* = 96)	Low-TyG-index group (*n* = 35)	*P*-value
Analysis length, mm				
Baseline	12 (10, 18)	13 (10, 20)	12 (9, 15)	0.637
Follow-up	12 (10, 18)	13 (10, 20)	11 (9, 15)	0.572
Vessel volume, mm^3^				
Baseline	191 (120, 282)	205 (129, 284)	171 (86, 259)	0.286
Follow-up	181 (108, 277)	196 (116, 285)	256 (81, 256)	0.266
Change from baseline	−4 (−14, 3)	−2 (−13, 4)	−9 (−19, −3)	0.015
*p*-value for change	0.583	0.668	0.694	
Plaque volume, mm^3^				
Baseline	93 (58, 144)	97 (67, 144)	79 (51, 147)	0.402
Follow-up	95 (57, 143)	100 (63, 141)	80 (49, 152)	0.445
Change from baseline	−1 (−7, 6)	0 (−5, 5)	−3 (−10, 8)	0.567
*p*-value for change	0.817	0.829	0.967	
PAV, %				
Baseline	48 ± 10	49 ± 9	46 ± 11	0.079
Follow-up	47 ± 10	48 ± 10	44 ± 10	0.049
Change from baseline	−1.2 ± 4.8	−1.5 ± 5.8	−1.0 ± 4.3	0.588
*p*-value for change	0.335	0.445	0.547	

PAV, percentage atheroma volume; TyG index, triglyceride-glucose index.

### 3.3 Association with the baseline TyG index and plaque progression

**Figure 2:**
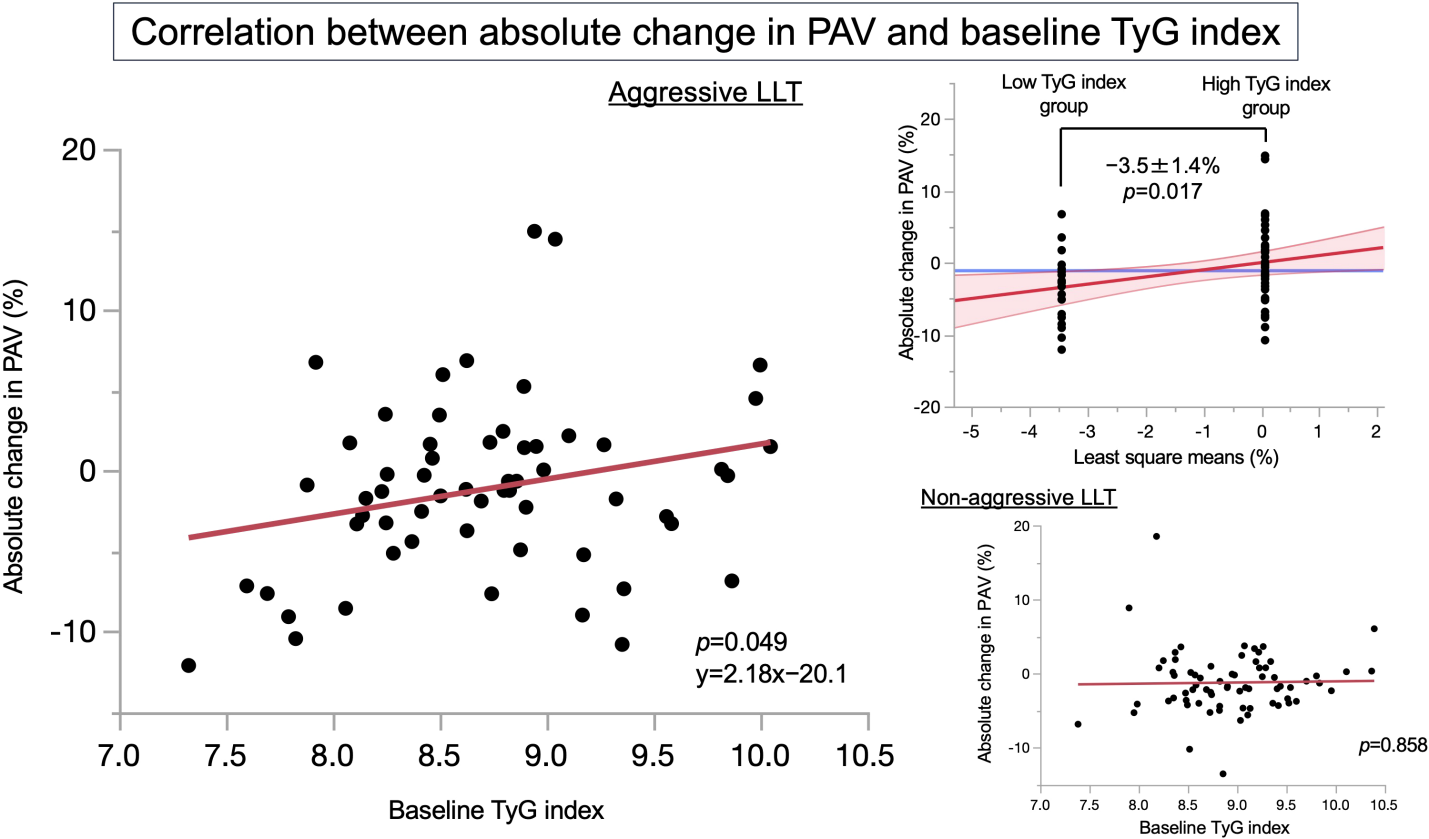
Correlations between absolute change in PAV and the baseline TyG index. This figure has shown the correlations between absolute change in PAV and the baseline TyG index in patients who received aggressive LLT (left panel) and in patients who did not receive aggressive LLT (lower right panel), respectively. In addition, the upper right panel has shown the result of analysis of covariance for absolute change in PAV between the low-TyG-index group and the high-TyG-index group only in patients who received aggressive LLT. LDL-C, low-density lipoprotein cholesterol; LLT, lipid-lowering therapy; PAV, percentage atheroma volume; TyG index, triglyceride-glucose index.

**Figure 2** shows the correlation between absolute change in PAV and the baseline TyG index according to the presence or absence of aggressive LLT. Among the patients who received aggressive LLT, there was significant correlation with absolute change in PAV and the baseline TyG index (*p* = 0.049), whereas there was no significant correlation between these in patients who did not receive aggressive LLT. In addition, the absolute change in PAV between the low-TyG-index group and high-TyG-index group only in patients who received aggressive LLT was −3.5% ± 1.4% by analysis of covariance (*p* = 0.017). Furthermore, **Table 3** shows the multiple linear regression analysis predicting the absolute change in PAV under aggressive LLT. As a result, baseline TyG index (estimate 2.71; 95% CIs 0.43, 5.00; *p* = 0.007), male (estimate 2.97; 95% CIs 0.32, 5.62; *p* = 0.029), and %change in LDL-C (estimate 0.102; 95% CIs 0.006, 0.197; *p* = 0.037) were significantly associated with the absolute change in PAV.

**Table 3:** Multiple linear regression analysis predicting the absolute change in PAV under aggressive LLT.

Explanatory variables	Simple linear regression			Multiple linear regression		
	Estimate	95% CIs	*P*-value	Estimate	95% CIs	*P*-value
Baseline TyG index	2.18	0.00, 4.35	0.049	2.71	0.43, 5.00	0.021
Male	1.75	0.56, −4.05	0.134	2.97	0.32, 5.62	0.029
%change in LDL-C	0.08	0.02, 0.14	0.009	0.102	0.006, 0.197	0.037
Acute coronary syndrome	−1.48	−3.10, 0.14	0.073	1.34	−1.26, 3.94	0.305
Age	−0.01	−0.15, 0.13	0.940	0.06	−0.10, 0.22	0.451
%change in BMI	−0.01	−0.09, 0.08	0.900	−0.03	−0.12, 0.06	0.474
Absolute change in HbA1c	0.31	−1.44, 2.06	0.722	0.63	−1.23, 0.06	0.501

BMI, body mass index; HbA1c, hemoglobin A1c; LDL-C, low-density lipoprotein cholesterol; LLT, lipid-lowering therapy; PAV, percentage atheroma volume; TyG index, triglyceride-glucose index.

## 4 Discussion

The major findings of this study are as follows: (1) This study verified the impact of the TyG index on coronary artery plaques detected by serial IVUS in patients with CAD under LLT represented by statins. (2) Compared with the low-TyG-index group, the high-TyG-index group was significantly associated with metabolic dysfunction, such as high BMI and triglyceride levels, DM, and low HDL-C levels. (3) Moreover, the absolute change in PAV between the two groups only in patients who received aggressive LLT was −3.5% ± 1.4% by analysis of covariance, and the baseline TyG index in these patients was significantly associated with the absolute change in PAV by multiple linear regression analysis. In addition, if the patients were divided into two groups according to the cutoff value of the baseline TyG index (dichotomized to ≥ or <8.4, or dichotomized to ≥ or <8.3, respectively) based on the interpretation of the sensitivity analysis, analysis of covariance revealed that the absolute change in PAV between the low-TyG-index group and the high-TyG-index group only in patients who received aggressive LLT was −3.5% ± 1.5% (*p* = 0.023) and −3.5% ± 1.6% (*p* = 0.034), respectively. The finding was consistent with a sensitivity analysis performed to confirm the robustness of the analytical method used in this study.

It has been reported that insulin resistance contributes to the development of cardiovascular disease in patients without DM and in those with DM (32), and is also involved in the development of several metabolic disorders and poor outcomes of cardiovascular disease (33). The reason for this is that insulin resistance not only induces inflammation and oxidative stress due to glucose metabolic imbalance, but also causes systemic lipid disturbances such as elevated triglyceride, small dense LDL, and postprandial excess lipid levels as well as reduced HDL-C levels, thus impairing endothelial function (34–36). This study has shown that the high TyG index is associated with a higher prevalence of cardiometabolic risk factors, such as obesity/high BMI, dyslipidemia (high triglyceride levels and low HDL-C levels), high fasting plasma glucose, and HbA1c. In addition, insulin resistance could contribute to not only both thrombosis and inflammation caused by platelet hyperactivity but also increased diastolic left ventricular stiffness and cardiac fibrosis caused by smooth muscle cell proliferation, collagen crosslinking, collagen deposition, and dysfunctional perivascular adipose tissue (37–39). Therefore, the TyG index could be a surrogate marker for the progression of atherosclerosis due to metabolic flexibility, endothelial dysfunction, coagulation disorders, and smooth muscle cell dysfunction (40).

This study is the first to report the impact of the baseline TyG index on change in coronary plaque volume assessed by IVUS. As a result, the baseline TyG index tended to be positively correlated with baseline PAV (*p* = 0.066) and was significantly associated with the absolute change in PAV only in patients receiving aggressive LLT. Thus, if the baseline TyG is already high at the time of onset of CAD, the coronary plaque burden tends to be high, and the progression of atherosclerosis may potentially play a strong role, even in patients subsequently receiving aggressive LLT. Thus, the higher the risk of cardiovascular events, the more stringent secondary prevention is required.

A recent study has reported that the higher long-term trajectory of the TyG index and the higher baseline TyG index were significantly associated with an increased risk of incident cardiovascular disease events and all-cause mortality in the future (41); thus, we need to discuss the association between the trajectory of the TyG index and coronary plaque burden. This study showed that the mean TyG index rose from 8.8 at baseline to 9.4 at the time of follow-up; however, there was no significant relationship between %change in the TyG index and absolute change in PAV, even in patients who achieved the target LDL-C value. Based on the literature review, the mechanism linking TyG index changes with coronary plaque progression seems reasonable. The time-dependent increase in the TyG index may lead to a greater accumulation of oxidative stress due to hypertriglyceridemia, thereby causing endothelial dysfunction and contributing to the initiation and progression of atherosclerosis (42). Additionally, prolonged exposure to hyperglycemia may promote the glycation of platelet proteins, enhance platelet reactivity, and potentially develop coronary plaques (43). In this study, **Table 4** shows that the %change in the TyG index was significantly associated with the absolute change in HbA1c (estimate 3.08; 95% CIs 1.64, 4.51; *p* < 0.001), %change in LDL-C (estimate 0.06; 95% CIs 0.01, 0.10; *p* = 0.008), and ACS (estimate 1.37; 95% CIs 0.17, 2.58; *p* = 0.026), but not age, %change in BMI, and male. Only seven patients had both exacerbation of HbA1c and increase in LDL-C levels, and these influencing factors for the change in the TyG index were relatively stable; thus, the involvement of lifestyle factors and dietary habits should be considered. The importance of lifestyle interventions was supported by data from the Brazilian cohort study (44), and then a meta-analysis revealed the impact of bariatric surgery on the TyG index, indicating improved insulin sensitivity and metabolic health (45). In addition, the effect of novel anti-diabetic drugs, such as glucagon-like peptide-1 receptor agonists and sodium-glucose co-transporter-2 inhibitors, on the TyG index has been shown (46, 47). Although the improvement in %change TyG index by these therapies is expected, whether these interventions necessarily contribute to coronary plaques is controversial. Among patients with CAD, the patients with ACS have more pre-existing atherosclerosis risk factors and are potentially more likely to increase the TyG index over time. However, the strict lipid management with LLT for patients with ACS could lead to greater plaque regression (48). The aggressive LLT was performed in approximately half of the patients in this study, which may have resulted in differences in the occurrence of cardiovascular events at the patient level and the degree of atherosclerosis development at the plaque level.

**Table 4:** Multiple linear regression analysis predicting the %change in TyG index.

Explanatory variables	Simple linear regression			Multiple linear regression		
	Estimate	95% CIs	*P*-value	Estimate	95% CIs	*P*-value
Absolute change in HbA1c	2.94	1.51, 4.38	<0.001	3.08	1.64, 4.51	<0.001
%change in LDL-C	−0.008	−0.023, 0.007	0.281	0.06	0.01, 0.10	0.008
Acute coronary syndrome	0.23	−0.86, 1.33	0.674	1.37	0.17, 2.58	0.026
Age	0.06	−0.04, 0.17	0.403			
%change in BMI	0.09	0.02, 0.16	0.008			
Male	0.04	−1.91, 1.98	0.971			

BMI, body mass index; HbA1c, hemoglobin A1c; Hs-CRP, high-sensitivity C-reactive protein; LDL-C, low-density lipoprotein cholesterol; TyG index, triglyceride-glucose index.

### 4.1 Limitations

This study had several limitations that require consideration. First, unknown confounding factors may have affected the study outcomes regardless of the analytical adjustments, and the relatively small number of enrolled patients may have limited the statistical power of the study. Second, this study was limited to patients with stable CAD and ACS who underwent percutaneous coronary intervention for culprit lesions and were analyzed for non-culprit lesions. Thus, the validity of this causal relationship in healthy individuals and patients eligible for primary prevention is unclear. Third, this study only included Japanese patients, and the results and effects may differ between races. Fourth, we need to note heterogeneity as a limitation of this study. (1) Clinical heterogeneity: **Supplementary Table 1** shows the comparison of baseline patients’ characteristics in three studies. The ENTERPRISE trial targeting stable patients with CAD had the highest BMI, triglyceride, and proportion of hypertension, and the lowest HDL-C and LDL-C. In the ESPECIAL-ACS study targeting patients with ACS, all patients were men and had DM, and the baseline TyG index was the highest. The ZEUS trial had the highest proportion of achieved aggressive LLT. (2) Methodological heterogeneity: It was unclear whether LLT was initiated recently or had been administered for many years. A total of 21 out of 44 patients in the ENTERPRISE trial and all patients in the ZEUS trial had recently received LLT, whereas details regarding when LLT was initiated were unclear in the ESPECIAL-ACS study. In addition, the follow-up period for the ENTERPRISE trial was 1 year, but for the ESPECIAL-ACS study and ZEUS trial, it was 6 months. (3) Statistical heterogeneity: Despite the above heterogeneity, the outcomes of all studies were standardized as the absolute change in PAV, and no significant variation in outcomes between studies was observed, even in patients who achieved aggressive LLT. Fifth, lifestyle factors and dietary habits are associated with coronary plaques as well as the TyG index; however, there were no data of studies on physical inactivity, heavy alcohol consumption, and diets high in carbohydrates, sugars, and red meat, and low in fruit and vegetable intake.

## 5 Conclusions

The aim of this study was to evaluate the impact of the TyG index on coronary artery plaques detected by serial IVUS in patients with CAD under LLT represented by statins. Among the patients who received aggressive LLT, the high-TyG-index group had a tendency for coronary plaque progression. In addition, multiple linear regression analysis revealed that the baseline TyG index was significantly positively associated with the absolute change in PAV. Thus, an elevated TyG index could be a residual risk in coronary plaque progression under aggressive LLT.

## Data Availability

The original contributions presented in the study are included in the article/**Supplementary Material**. Further inquiries can be directed to the corresponding author.
