# Supplementary material for: Impact of TyG index on coronary plaques in patients with coronary artery disease under aggressive lipid-lowering therapy
Source: Front Endocrinol (Lausanne). 2026 Feb 12;17:1766778. doi: 10.3389/fendo.2026.1766778 (PMC12935650; doi:10.3389/fendo.2026.1766778)
Supplement: Supplementary file 1 [file Table1.docx]

Supplementary Table 1. Comparison of baseline patients’ characteristics in three studies

| Variables | ENTERPRISE trial  (n=44) | ESPECIAL-ACS study  (n=35) | ZEUS trial  (n=52) | *p* value |
| --- | --- | --- | --- | --- |
| Age, years | 62±8 | 64±8 | 63±13 | 0.684 |
| Male, n (%) | 43 (98) | 35 (100) | 42 (81) | 0.001 |
| Body mass index, kg/m^2^ | 27.1±3.1 | 24.6±3.1 | 23.6±3.6 | <0.001 |
| Hypertension, n (%) | 41 (93) | 24 (71) | 37 (71) | 0.009 |
| Diabetes mellitus, n (%) | 27 (61) | 35 (100) | 15 (29) | <0.001 |
| TyG index | 8.9±0.6 | 9.1±0.5 | 8.6±0.6 | <0.001 |
| Triglyceride, mg/dL | 162±98 | 133±65 | 106±48 | 0.001 |
| HDL-C, md/dL | 41±10 | 44±9 | 50±13 | <0.001 |
| LDL-C, md/dL | 79±23 | 108±30 | 116±24 | <0.001 |
| Achieved aggressive LLT, n (%) | 14 (32) | 6 (17) | 39 (59) | <0.001 |
| Acute coronary syndrome, n (%) | 0 (0) | 35 (100) | 52 (100) | <0.001 |

HDL-C, high-density lipoprotein cholesterol; LDL-C, low-density lipoprotein cholesterol; LLT, lipid-lowering therapy; TyG index, triglyceride-glucose index.
